# The effectiveness and feasibility of TREAT (Tailoring Research Evidence and Theory) journal clubs in allied health: a randomised controlled trial

**DOI:** 10.1186/s12909-018-1198-y

**Published:** 2018-05-09

**Authors:** Rachel J. Wenke, Rae Thomas, Ian Hughes, Sharon Mickan

**Affiliations:** 1 Ground floor Allied Health Services, Gold Coast Health, Gold Coast University Hospital, 1 Hospital Boulevard, Southport, Qld 4215 Australia; 2 School of Allied Health Sciences, Griffith University, Southport, QLD 4215 Australia; 3 Centre for Research in Evidence Based Practice (CREBP), Bond University, Robina, QLD 4229 Australia

**Keywords:** Journal club, Allied health, Evidence-based practice

## Abstract

**Background:**

Journal clubs (JC) may increase clinicians’ evidence-based practice (EBP) skills and facilitate evidence uptake in clinical practice, however there is a lack of research into their effectiveness in allied health. We investigated the effectiveness of a structured JC that is Tailored According to Research Evidence And Theory (TREAT) in improving EBP skills and practice compared to a standard JC format for allied health professionals. Concurrently, we explored the feasibility of implementing TREAT JCs in a healthcare setting, by evaluating participating clinicians’ perceptions and satisfaction.

**Methods:**

We conducted an explanatory mixed methods study involving a cluster randomised controlled trial with a nested focus group for the intervention participants. Nine JCs with 126 allied health participants were randomly allocated to receive either the TREAT or standard JC format for 1 h/month for 6 months. We conducted pre-post measures of EBP skills and attitudes using the EBP questionnaire and Assessing Competence in Evidence-Based Medicine tool and a tailored satisfaction and practice change questionnaire. Post-intervention, we also conducted a focus group with TREAT participants to explore their perceptions of the format.

**Results:**

There were no significant differences between JC formats in EBP skills, knowledge or attitudes or influence on clinical practice, with participants maintaining intermediate level skills across time points. Participants reported significantly greater satisfaction with the organisation of the TREAT format. Participants in both groups reported positive changes to clinical practice. Perceived outcomes to the TREAT format and facilitating mechanisms were identified including the use of an academic facilitator, group appraisal approach and consistent appraisal tools which assisted skill development and engagement.

**Conclusions:**

It is feasible to implement an evidence-based JC for allied health clinicians. While clinicians were more satisfied with the TREAT format, it did not significantly improve their EBP skills, attitudes, knowledge and/or practice, when compared to the standard format. The use of an academic facilitator, group based critical appraisal, and the consistent use of appraisal tools were perceived as useful components of the JC format. A structured JC may maintain EBP skills in allied health clinicians and facilitate engagement, however additional training may be required to further enhance EBP skills.

**Trial registration:**

ACTRN12616000811404 Retrospectively registered 21 June 2016.

**Electronic supplementary material:**

The online version of this article (10.1186/s12909-018-1198-y) contains supplementary material, which is available to authorized users.

## Background

Clinical practice informed by research evidence is an important pre-requisite for health professionals to deliver quality patient outcomes [1, 2]. Allied health professionals, who make up the third largest healthcare clinical workforce, commonly report barriers to providing evidence based practice (EBP) including knowledge and confidence gaps, and lack of time [3–5]. Journal clubs (JC) describe a group of individuals who meet regularly to critique and discuss research articles, and are recognised as a tool to increase clinicians’ knowledge and use of research evidence in clinical practice [3, 6–8]. However, there is a lack of research exploring the effectiveness of JCs by allied health clinicians working in healthcare services.

Recent systematic reviews indicate that the majority of research exploring the effectiveness of JCs involves medical professionals [6, 9]. While heterogeneity among studies restricted meta analyses in these reviews, previous randomised controlled trials have demonstrated increased self-reported knowledge in medical interns [10], and objective critical appraisal skills in surgeons [11] who participated in a JC compared to control groups. Most research about the effectiveness of JCs within allied health includes case-based studies or small uncontrolled study designs [3, 7, 12–14]. The largest study evaluating the effectiveness of JCs in allied health, recruited 93 clinicians across five professional groups (physiotherapy, speech pathology, nutrition, occupational therapy and social work) to participate in a structured journal club model based on principles of adult learning and a collaborative approach between researchers and clinicians [7]. Following the six-month trial, the results suggested significant improvements in objective and self-reported measures of EBP knowledge, as measured by the Adapted Fresno Test and EBP uptake scale, with some professional groups also reporting increased evidence uptake and improved attitudes towards EBP [7]. While the uncontrolled study design was acknowledged as a limitation, other researchers have recognised that randomised controlled designs can be challenging to implement in educational and translational research [15]. Further, qualitative methodologies have beeen recommended to supplement randomised study designs, to assist with explaining results [9]. It is also important that future research evaluates JCs that are grounded in existing research and theory, and the goals of any intervention are tailored to the specific organisational and/or professional context [7, 9, 15].

### Local context

JCs are currently the most frequently used intervention to improve allied health clinicians’ knowledge and skills in EBP within Gold Coast Health (GCH), Australia. A group of local EBP champions consisting of approximately 12 GCH allied health clinicians conducted a quality improvement project with the guidance of authors RW and SM to evaluate local JC processes. The project identified substantial variation in both the implementation of JCs and their impact on individuals’ knowledge and use of research evidence in clinical services. In response, we synthesised the research evidence for effective implementation of JCs from two recent systematic reviews of 12 and 18 studies [6, 9]. This synthesis revealed 11 “key components” for effective JC implementation including having an overarching goal or purpose, support from researchers, a facilitator to guide discussion, adhering to principles of adult and multi-faceted learning, and evaluating knowledge uptake [6, 9]. An audit of existing JCs identified that most allied health JCs within GCH incorporated relatively few of these key components as a routine part of their JC sessions [6, 9].

### Aim

We aimed to evaluate the effectiveness and feasibility of implementing a JC, informed by the best research evidence and theory for allied health clinicians [6, 9]. Eleven key components identified in the research evidence were incorporated into a structured JC called “TREAT” (Tailoring Research Evidence and Theory), as shown in Table 1. We evaluated the effectiveness of the TREAT JC format on improving clinicians’ EBP skills, knowledge, attitudes and practice compared with the standard JC format for allied health professionals. We also sought clinicians’ satisfaction and perceptions of the TREAT JC format to explore feasibility of implementation.

**Table 1:** Components of TREAT journal club format

Component from evidence	Consistently conducted in standard?	Local modification
1. Establish JC of similar interests ^	✔	- JC participants from similar clinical background or interest - Initial goal setting session to establish topics of interest to all members that will be discussed in journal club.
2. Have overarching goal and purpose ^	☓	As above.
3. Regular predictable attendance ^	✔	Journal club set at same time and location each month
4. Circulating articles for discussion ^	☓	Journal articles circulated prior to journal club
5. Didactic support ^	☓	Didactic teaching initially provided within each session on given topic by research academic and later given as handouts for reference^a^
6. Mentoring/Support from researchers/academics ^^a^	☓	Academic facilitator available for support between sessions
7. Have a facilitator to guide discussion^	☓	Academic facilitator helped guide discussions during each session
8. Use of structured appraisal tools during the session ^^a^	☓	Standardised critical appraisal tool used (Critical Appraisal Skills Programme)
9. Adhering to principles of adult learning and use multi-faceted learning strategies^a^	☓	-Group approach to critical appraisal to promote collaborative learning-Incidental teaching based on participant motivations within the session- Written based resources and access to library support to assist with searching
10. Put evidence in context of clinical practice and evaluate knowledge uptake informally or formally ^	☓	Time provided in session to discuss clinical implications and follow up of knowledge uptake.
11. Provide food^	☓	JC club participants invited to bring food to share for session

^= key component suggested in Deenadayalan et al., 2008 ^a^ = key component suggested in Harris et al., 2011

^a^Due to time constraints this teaching was provided in the form of a handout in later sessions

## Methods

### Study design

We conducted an explanatory sequential mixed methods project to address both implementation and intervention objectives. A cluster randomised controlled trial was used to investigate changes in allied health clinicians’ EBP skills, knowledge, attitudes and practice and a nested focus group captured clinicians’ perceptions of the TREAT JC format. Ethical approval for the study was provided (HREC/15/QGC/310) prior to commencement. The trial was also retrospectively registered 21 June 2016 (ACTRN12616000811404).

### Participants

**Fig. 1:**
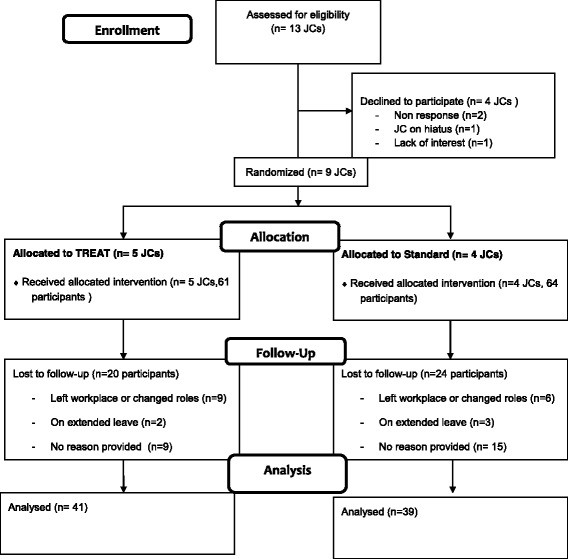
Participant flow through study

After seeking approval from line managers, we invited all 13 existing allied health JCs within GCH to participate. Nine JCs agreed to participate and were randomised to receive either the TREAT JC format or to continue with their standard JC format (see Fig. 1). Written informed consent was obtained from all participants. Authors identified the JCs as “small” (6–15 attendees) or “large” (> 16 attendees) and this difference in size was used to stratify randomisation. An independent researcher used a computer generated random block design to allocate JCs (in each group) to TREAT or Standard. Allocations were concealed in opaque envelopes numbered sequentially (1 to 5 “small” and 1 to 5 “large”). These envelopes were opened by the JC participants of each group after their pre-assessment sessions. To maximise variation of professional experience and JC attendance, we purposively sampled 61 participants from the TREAT format to invite 18 clinicians to participate in focus groups. Eight clinicians consented to attend from four different TREAT JCs.

### Intervention

All JCs were encouraged to continue meeting for their one-hour, monthly JCs, at their nominated time and venue for the six-month intervention period. Five JCs were randomly allocated to the TREAT format, which incorporated eleven key components of successful journal clubs (see Table 1). These key components were tailored in consultation with a group of allied health EBP champions across GCH to optimise operational feasibility. The TREAT JCs were facilitated by academic allied health researchers experienced in teaching and using EBP (RT, 20/30 JCs, RW 6/30 and SM 4/30 JCs). Each JC followed a consistent format (see Additional file 1) including: initial goal setting to identify relevant topics; use of the PICO approach to clarify clinical questions, group critical appraisal using structured “Critical Appraisal Skills Programme” [16] tools; engaging librarian support; and formally documenting actions. Clinicians allocated to the standard group were asked to continue using their JC format as they had previously done for the duration of the trial. In contrast to the TREAT format, this generally consisted of a clinician choosing an article of interest, appraising it themselves (with or without the use of a formal appraisal tool) and presenting it to the rest of the group without any formal facilitation or follow up. Clinicians in both TREAT and standard JCs completed an assessment of their EBP knowledge and skills at baseline and immediately after the six-month JC intervention. Adherence and adaptation [17] to the TREAT format were monitored monthly by the research team. Due to the nature of the intervention, blinding of participants was not possible however the use of both objective and subjective outcome measures helped to reduce any bias associated with known treatment allocation.

### Materials

#### The evidence based practice questionnaire (EBPQ)

The EBPQ is a 24-item, self-report questionnaire used to assess an individual’s practice, attitudes towards and knowledge of EBP [18]. Responses to items are recorded on a seven point Likert scale with higher scores indicating a more positive attitude towards EBP [18]. The practice behaviour subscale asks respondents how frequently they practice the five phases of EBP and how frequently they shared the EBP information with colleagues. EBP attitudes are measured with four statements where respondents are asked to nominate which statement is most like them. Finally, self-reported EBP knowledge is assessed by 14 items where respondents are asked to nominate how they would rate themselves on key tasks. There are no reported cut offs for this measure. In a nursing population, the EBPQ has been found to have high internal consistency (α = 0.87) with subscale internal consistency ranging from α = 0.79 to α = 0.91 [18]. Moderate yet positive construct validity of this questionnaire has been reported, with correlation coefficients ranging between 0.3 and 0.4 [18].

#### Assessing competence in evidence-based practice (ACE tool)

The ACE tool is a 15-item measure of applied EBP skills to a hypothetical clinical scenario, literature search and results [19]. Items are responded to dichotomously, and are grouped into four of the five steps in evidence-based practice [19]. Three medical trainee cohorts were used to measure construct validity and resulted in statistically significant trends corresponding to level of training. ACE Total score for EBM (evidence based medicine)- novice was 8.6 ± 2.4, EBM-intermediate was 9.5 ± 1.8 and EBM advanced 10.4 ± 2.2. Cronbach’s alpha for internal consistency was 0.69 [19].

After the JC intervention, participants completed a purpose-designed self-report questionnaire, which explored their satisfaction with the JC and how their clinical practice was influenced after participating in each JC session (see Additional file 2). For the influence on clinical practice component of the questionnaire, clinicians were asked to nominate whether they attended each JC and whether the topic was of clinical relevance to their practice. For those JCs attended and relevant, clinicians were then asked to rate (five-point Likert scale) how strongly they agreed that the article discussed either changed or confirmed their current practice. The higher rating on either Likert scale (changed or confirmed) was used for analyses. Where a change in practice was reported, clinicians were asked to identify the type of change (e.g., adopting new guideline, treatment strategy).

### Clinician focus group

Following the JC intervention, focus groups with participants from the TREAT JC were conducted to gain a deeper insight into the clinician’s experiences. Themes from the purpose designed clinician satisfaction questionnaire were used to inform probing questions for the focus group. Two 1-h focus groups were conducted by independent facilitators using a semi-structured interview guide (Additional file 3).

### Data analyses

Randomisation occurred at the group level and data analyses were performed at the individual level. Being a pilot research project, we recruited a convenience sample based on the existing number of journal clubs within the recruitment site and an estimated 80% consent rate.

#### Quantitative analysis

Between group differences for the EBPQ and ACE tool were analysed using mixed effects models with random effects being JC cluster and individual participant and fixed effects including group*prepost interaction to test the effect of TREAT versus standard JCs over time. Covariates in the model included attendance, age, gender, profession, possession of a research higher degree, and level of clinical experience. JC cluster was found to contribute less than 10% to the total variation in all cases so the final mixed effects analyses used individual as the only random effect. For outcome measures that were only administered post intervention (i.e., satisfaction questionnaire and influence on practice ratings), simple least squares regression or logistic regression were used as required with group as the variable of interest. As the impact of covariates was not an aim of the study, significant results (*p* = 0.05) only will be reported. To explore specific skills of the ACE tool more closely, item level responses to the ACE tool questions were also analysed descriptively. Dummy codes were allocated to evaluate changes between correct and incorrect answers before and after the intervention. All quantitative analyses were conducted using a per protocol analysis with no imputation of missing data.

#### Qualitative analysis

Data were gathered from open ended questions within the satisfaction questionnaires and focus group transcripts. Two levels of analyses were undertaken which included a thematic description of the data and then an interpretative thematic analysis looking for latent themes [20]. For the first level of analyses, we used content analysis to develop initial categories from the questionnaires. Focus group transcripts were then coded by one of the authors (RW), with formation of categories and sub-categories being based on semantic meanings of the data with discussion and checking with SM. A second level of thematic analyses and synthesis was then conducted by RW to identify explanatory themes from the data of participants taking part in the TREAT format which were checked and discussed with SM. In this, inductive coding was used to help examine the underlying ideas, assumptions and conceptualisations in the data [20].

#### Mixed methods interpretation

Both quantitative and qualitative data were analysed independently. They were brought together for interpretation after these analyses.

## Results

Nine journal clubs participated in the trial. Pre-assessment surveys were completed by 126 clinicians and 80 (~ 64%) completed both pre- and post-assessment questionnaires. Reasons for loss at follow up are provided in Fig. 1 and included participants moving to another work role (*n* = 15) or being on extended leave (*n* = 5) or no reason given (*n* = 25) (i.e., participants not responding to contact made by researchers). No significant differences on baseline total scores of the ACE tool or EBPQ, age, gender, practice setting, and years of clinical experience were found between those who did and did not complete the post assessment. There was a significant difference between the groups for profession (*p* = 0.03) and proportion of people completing higher degrees (*p* = 0.048), with about 10% more participants who completed a research higher degree in the drop out group, and less speech pathologists, physiotherapists and more pharmacists in the drop out group.

There were no differences between the two groups regarding demographic variables or pre-assessment measures (see Tables 2 and 3). The majority of participants were female (106/125 85%), aged between 20 and 29 years (47/125, 38%) or 30 and 39 years (45/125, 36%), and with just under half the participants having between 2 and 5 years (29/125, 23%) or 5 and 10 years (30/125, 24%) clinical experience. Attendance at the six journal club sessions ranged from 0 to 6 sessions with an average attendance of 3.8 sessions. Participants in the TREAT focus groups ranged in their clinical experience and included base grade clinicians (*n* = 3), senior clinicians (*n* = 4) and one team leader with representation from four out of the five journal clubs across four professions (as invited participants from JC 1 participants were unavailable). Topics discussed at each journal club are presented in Additional file 4. Due to unforeseen service changes, one journal club in the standard group only had four JC meetings across the six months.

**Table 2:** Participant Demographic Information

		TREAT (*n* = 61) Freq. (%)	Standard (*n* = 64) Freq. (%)
Gender^a^	Male	12 (19.7)	6 (9.4)
	Female	49 (80.3)	57 (89.1)
Age Range^a^	20–29	17 (27.9)	30 (46.9)
	30–39	25 (41.0)	20 (31.3)
	40–49	11 (18.0)	5 (7.8)
	50–59	8 (13.1)	6 (9.4)
	60–69	–	2 (3.1)
Allied Health Profession	Dietician	23 (37.7)	2 (3.1)
	Social Worker	1 (1.6)	1 (1.6
	Psychologist	2 (3.3)	–
	Occupational Therapist	8 (13.1)	6 (9.4)
	Speech Pathologist	–	23 (35.9)
	Physiotherapist	11 (18.0)	2 (3.1)
	OT Assistant	3 (4.9)	2 (3.1)
	Nurse	9 (14.8)	5 (7.8)
	Allied Health Assistant	1 (1.6)	1 (1.6)
	Podiatrist	1 (1.6)	–
	Exercise Physiologist	1 (1.6)	–
	Pharmacist	1 (1.6)	22 (34.4)
Clinical Experience (Yrs)^a, b^	< 2	7 (11.5)	18 (28.1)
	2–5	14 (23.0)	15 (23.4)
	5–10	18 (29.5)	12 (18.8)
	10–15	10 (16.4)	8 (12.5)
	> 15	11 (18.0)	10 (15.6)
Higher Research^c, d^	None	53 (86.9)	51 (79.7)
	Graduate Diploma	–	5 (7.8)
	Honours	2 (3.3)	2 (3.1)
	Masters of Research	–	2 (3.1)
	Masters + PhD	1 (1.6)	–
	Masters – Other	2 (3.3)	1 (1.6)
	Post Graduate Cert	1 (1.6)	–

^a^Standard *n* = 63; ^b^TREAT *n* = 60; ^c^TREAT *n* = 59; ^d^Standard n = 61

**Table 3:** Comparison between TREAT and Standard Journal Club pre- to post-intervention

Test item	Pre-Intervention			Post-Intervention		
	Group (*N*)	M	95% CI	M	95% CI	Between group effects
						*P* value
EBPQ (max score=)						
Practice (max = 42)	TREAT (40)	26.5	24.6–28.5	26.5	24.6–28.5	.314
	Standard (40)	26.9	24.9–28.8	27.9	25.9–29.9	
Attitudes (max = 28)	TREAT (40)	21.7	20.8–22.6	21.9	20.6–23.3	0.875
	Standard (40)	21.9	21.1–22.8	21.5	20.2–22.9	
Knowledge (max = 98)	TREAT (39)	63.8	61.2–66.4	68.1	65.7–70.5	0.167
	Standard (40)	66.2	63.6–68.8	66.9	64.6–69.3	
ACE Tool (max score=)						
Answerable Question (max = 2)	TREAT (41)	1.2	1.0–1.4	1.2	0.9–1.4	0.674
	Standard (39)	1.2	0.9–1.3	1.2	0.9–1.3	
Searching the literature (max = 2)	TREAT (41)	1.4	1.2–1.6	1.6	1.4–1.8	0.485
	Standard (39)	1.2	1.1–1.4	1.3	1.0–1.5	
Critical Appraisal (max = 7)	TREAT (41)	4.2	3.9–4.5	4.3	4.1–4.6	0.529
	Standard (39)	4.3	4.1–4.5	4.4	4.1–4.7	
Applying the evidence (max = 4)	TREAT (41)	2.4	2.2–2.6	2.2	1.9–2.3	0.341
	Standard (39)	2.3	2.1–2.5	2.3	2.1–2.5	
Total (max = 15)	TREAT (41)	9.2	8.8–9.5	9.3	8.8–9.7	0.608
	Standard (39)	9.0	8.6–9.5	9.1	8.6–9.6	

Some adaptations to the original TREAT JC format were made during the trial. Due to time constraints in the JC session, the use of didactic teaching was removed from the TREAT format after the first week and converted into a paper-based and electronic resource. While clinicians were invited to bring food along to their JC sessions, this did not consistently occur.

### EBP practice, attitudes and knowledge (EBPQ)

Overall, allied health clinicians scored slightly above the mid-point (TREAT *M* = 26.5, Standard *M* = 26.9 out of a possible 42) for self-reported EBP practice at pre-assessment and scores increased slightly at post-assessment on the EBPQ (Table 3). However, there was no difference between TREAT and Standard JC participants on EBP practice of the EBPQ at post-assessment*.* EBP attitudes were very positive at pre-assessment (TREAT *M* = 21.7, Standard *M* = 21.9 out of a possible 28) and remained so at post-assessment and there was no statistically significant difference between groups*.* Participant’s self-reported EBP knowledge was slightly above average at pre-assessment (TREAT JC *M =* 63.8, Standard JC *M =* 66.2 out of a possible 119) and there was no difference between groups at post-assessment. The mixed effect models revealed that profession type (p = < 0.05) gender (*p* = 0.038), clinical experience (*p* = 0.05) and whether the participant had a research higher degree (*p* = 0.003) significantly influenced certain items of the participant’s EBPQ ratings. There was no effect of group however as indicated by a non-significant interaction of group with time.

### EBP skills (ACE tool)

There were no significant between group differences in skills-based assessment of EBP (see Additional file 5 for individual item descriptive analyses for the ACE tool). Clinicians in both groups scored in the intermediate level at both pre- and post-assessment. There were no significant differences between groups on the four key steps of EBP before and after the intervention. While some of the variables including age, clinical experience and profession were significant predictors for certain items of the ACE tool (*p* = < 0.05), there was no significant interaction between group or time to indicate any effect of the TREAT JC compared to the standard JC.

### Satisfaction

All participants rated the JCs highly for overall satisfaction, usefulness and value (Table 4). However, the TREAT JC participants were significantly more satisfied with the organisation of the journal club, when compared with their standard JC participants. All participants would recommend journal clubs to other clinicians. When the mixed model accounted for clinical experience, there was also a significant difference between groups for the item pertaining to whether the JC should continue (*p* = 0.007), with the TREAT JC being rated more favourably. There was a significant effect of research higher degree type on perceptions of the JC being valuable (*p* = 0.024) however this did not have an effect on detecting any differences between the two groups.

**Table 4:** Post-Intervention comparison of satisfaction

				95% CI	Between group
Measure of Satisfaction	Group (N)	Mean rating (max = 5)	SD		*p value*
Usefulness	TREAT (41)	4.07	0.61	3.87–4.26	0.329
	Standard (39)	3.92	0.58	3.73–4.10	
Valuable	TREAT (41)	4.2	0.68	3.98–4.41	0.997
	Standard (39)	4.08	0.74	3.84–4.31	
Organisation	TREAT (41)	4.3	0.67	4.09–4.51	0.049**
	Standard (39)	3.9	0.7	3.67–4.12	
Should they Continue	TREAT (41)	4.2	0.68	3.98–4.41	0.08*
	Standard (39)	3.9	0.7	3.67–4.12	
Recommend to Others	TREAT (41)	4.2	0.82	3.94–4.45	0.371
	Standard (39)	4.08	0.58	3.89–4.27	

N/B ** = statistically significant. *When clinical experience was used in the mixed effects model, this was found to be significant (*p* = 0.007)

### Influence on clinical practice

Participants in both groups rated the JC as positively influencing their clinical practice across the sessions, with scores averaging above 3.5 out of 5 (Table 5). No significant difference between groups was found. The most frequently reported clinical practice change was updating a guideline or pathway and adopting new therapy strategies (Additional file 6). Fewer clinicians reported stopping therapy or initiating research or quality activity as a result of the JC session. A significant difference in the frequency of clinical practice changes was identified between groups for session 6, where significantly more clinicians reported adopting a new treatment strategy in the standard group compared to the TREAT group (*p* = 0.005). As this difference was likely impacted by a number of factors external to the JC format including the evidence being appraised for that week and how this was conducted, we cannot interpret this finding. No other significant differences between groups were identified.

**Table 5:** Clinician ratings of influence of journal club session on clinical practice

Journal Club session	Group (N)	Mean rating (max = 5)	SD	95% CI	Between group *p value*
Session 1	TREAT (28)	3.71	0.66	3.45–3.96	
	Standard (27)	3.81	0.68	3.54–4.08	0.823
Session 2					
	TREAT (28)	3.57	0.63	3.32–3.81	
	Standard (17)	3.70	0.69	3.34–4.05	0.453
Session 3					
	TREAT (18)	3.55	0.70	3.20–3.89	
	Standard (26)	3.84	0.61	3.59–4.08	0.707
Session 4					
	TREAT (21)	3.9	0.62	3.61–4.18	
	Standard (20)	3.65	0.67	3.33–3.96	0.305
Session 5					
	TREAT (16)	4.06	0.68	3.69–4.42	
	Standard (19)	3.78	0.85	3.37–4.18	0.362
Session 6					
	TREAT (26)	3.81	0.80	3.48–4.13	
	Standard (17)	4.00	0.50	3.74–4.25	0.223

### Qualitative categories and themes

Initial descriptive analyses of the open-ended questionnaire responses (both groups) and focus group data (TREAT group only) revealed four main categories that reflected the feasibility of this intervention: 1) outcomes of the JC, 2) facilitating mechanisms 3) challenges and 4) suggestions for improvement. Within these themes, some differences were identified between the TREAT and Standard JC as shown in Table 6. For example, TREAT JC participants reported some different individual outcomes compared to participants in the Standard JC. This included an increased knowledge of and confidence in critical appraisal and changes to clinical practice including developing a new pathway of clinical care, “*we got a new pathway and we were able to do a quality activity and write up and circulate it with our colleagues…so that was really great”* [F2]. The TREAT format was also reported to result in greater time efficiency, *“so it[appraisal] was done as a group….where as previously the [presenter] would have … pre-prepared the appraisal so I think it was more time efficient”* [F1].

**Table 6:** Summary of qualitative themes from questionnaire and focus group

Category	SharedSubcategory	TREAT JC subcategories *(from focus group + questionnaire)*	Freq. of mention	Standard JC subcategories *(from questionnaire only)*	Freq. of mention
Outcomes	*Individual level*	Increased skills in appraisal	30	Increase/maintain knowledge across clinical areas	12
		Question how you use research	11	Aware of other professional interests or views	7
		More confidence in appraisal	3	Evidence being shared	9
		Attitudes towards research	3	Learning/applying critical appraisal	5
	*Service outcomes*	Changes or confirmed clinical practice	10	N/A	
		Potential to lead to other research	5		
		More time efficient	5		
Facilitating Mechanisms	*Structure/format of session*	Increased participation &MDT discussion	29	Roster, organisation of presenter in advance	5
		Six week structure encouraged attendance	10	Sending article early	4
		Appraisal tool and documentation	9	Clear appraisal tools	3
		Goal setting to identify relevant topics	9	Structured format	3
		Clinicians choosing topics in interest areas	6	Effort put in by presenters	3
		Less intimidating format	8	Having a portfolio for JC role	2
		Library support	4	*Support of team:*	
		*Benefit of Facilitator:*	21	Clinician’s research knowledge shared	6
		Incidental education	36	Enthusiasm/support of coordinating staff	4
		Increased confidence in outcome of appraisal	5	Getting staff advice on how to appraise	3
		*Specific mechanisms of facilitator:*		Positive team culture	2
		Insider knowledge & expertise	18	*Format for choosing article:*	
		Led deeper discussion	15	Linking article to clinical scenario/case	9
		Passion and energy to engage	5	Having article in area of interest	2
		Their availability	4		
		Preparedness	2		
*Challenges*	*Time & Staffing*	Attendance- time to come	38	Time to participate	13
		Reduced preparedness (not reading article beforehand)	23	Not enough time to review article beforehand	5
		Knowledge and skill barriers	3	Knowledge barriers	5
				Staff changes (attendance/restructuring)	4
	*Barriers of format*	Videoconference issues	9	Technology issue	2
		Minutes actions not always followed up	8	Unstructured and not useful format	2
		Too much time analysing article	6	Not enough appraising & discussing implications	2
		Powerpoints or handouts not given	2		
	*Evidence/Topic*	Lack of change in practice/ evidence to change	*9*	Reduced relevance of topic of article chosen	7
		Finding relevant topic for everyone	4		
	*Challenges to sustainability*	Turnover of staff	5	N/A	
		Forgetting what was learnt	3		
		Having access to mentor	3		
Suggestions for improvement	*Education/Support00*	Extra education	15	Academic support during club	6
		Teach someone else to be facilitator	4	EBP education	5
		Ongoing access to researcher for support	4		
	*Changes to format*	Have club at different time of day	5	Have more structured	5
		More time discussing application to practice	4	Ensure time to read article before/during session	4
		Choose topics as group at start	3	Discuss application to practice	3
		Ensure everyone reads article beforehand	3	Make more interactive	2

Freq. = Frequency. N/A = shared subtheme not applicable in participant responses

**Fig. 2:**
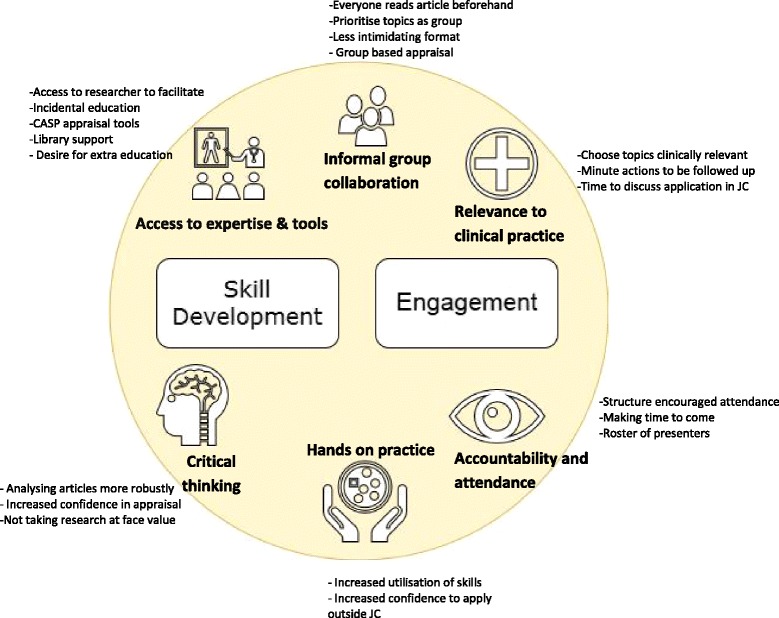
Thematic analysis of TREAT qualitative data

To further explore the feasibility of the TREAT JC from the clinician’s perspective, a second level of thematic analysis was undertaken. This analysis revealed two explanatory themes relating to the feasibility of the format which pertained to 1) skill development, and 2) engagement, as shown in Fig. 2 together with subthemes. Within the theme of skill development, clinicians reported access to expertise and tools was important. This included having access to the facilitator as part of the TREAT format, *“what everyone really appreciated was having an expert in the room to ask”* [F1]. Several attributes of the academic facilitator were described to contribute to the clinicians’ perceived value of the academic role in the TREAT format, including having a *“specific skill set… to impart that expertise to the person who’s asking the questions”* [F2], and *“keeping the flow and discussions happening”* [F1]. Other components reported to be useful for skill development included, “*access to library”* [F2], incidental education from the facilitators [F2] and use of CASP appraisal tools [Q3] to allow *“practice to analyse articles with more detail”* [Q9]. Extra education was however also suggested by clinicians which included, *“basic training… on how to interpret research articles, plot charts, p values etc.”* [Q5], and some kind of ongoing support or “*check-ups*” [Q1] with an academic facilitator. Within the theme of skill development, clinicians also reported using increased critical thinking, including analysing articles more robustly, *“we’re talking about things that we didn’t before. So we’re analysing articles in a more robust way*” [F1], and an increased desire and self-efficacy to use appraisal, “*it has given me the ability and desire to read, analyse, discuss and apply more”* [Q2] and “*I think by going through TREAT ….I’m much more critical and nit-picky… it shows you to really tear the article apart”* [F1]. Clinicians in the TREAT JC also felt the hands on practice of EBP skills within the JC session increased their utilisation of these skills, “*For me personally, I just got to utilise those skills a lot more*” and resulted in increased confidence to apply the skills outside of JC, “*I think just the actual practising of skills then made me confident to look at my own area”* [F1].

The second theme found in the data was related to engagement and included subthemes of informal group collaboration, accountability and attendance, and relevance to clinical practice. The collaborative group based appraisal were seen to promote engagement and be less intimidating, with clinicians reporting *“it was more collaborative and I guess less stressful for the person that’s actually brought that article to the group” *[F2]*,* with another clinician commenting, “*everyone seemed to (have) participated a lot more during TREAT than before or after”* [F1]. The prioritisation of topics as a group also facilitated engagement, *“the goal setting at the start of the six months just helped make sure we had something that everyone was interested in”.* [F1] Having everyone read articles beforehand was seen as a way to further enhance group engagement in the future [F1]. Within the subtheme of relevance to clinical practice, engagement was further promoted by *“choosing topics relevant to clinical practice”* [Q8], however this was a challenge at times for JCs who had members with diverse professions or interest areas attending [Q5], or where there was a lack of evidence on a certain topic “..*when you get to the end of it [article] and it’s not great and you ask the question are we going to implement this? No”* [F2]. Ensuring there was enough time to discuss the application of the evidence within the JC was also seen as important to application to practice, *“it would have been helpful if we’d spent a little bit more time on that, about how it can be applied in practice”* [F1].

A last subtheme of engagement was accountability and attendance. Clinicians reported the structure of the TREAT format encouraged attendance, *“because it was a six week [JC] block there was the intent of staff to attend. Whereas sometimes you can look at what’s being presented and choose whether you want to attend or not”* [F1]. Making time to come to the JC amongst “*competing priorities and clinical caseloads*” [F2] was however still considered a challenge to attending, with one TREAT participant reporting, “*while everybody recognises it [JC] as really important…the reason people are unable to attend is because something else more important is happening*” [F2].

## Discussion

This study is the first cluster RCT to evaluate the effectiveness and feasibility of an evidence-based JC intervention for allied health clinicians working in a healthcare setting. We demonstrated that it is feasible to deliver a structured and evidence-based JC with allied health clinicians. Participants in the TREAT JC were significantly more satisfied with the organisation of the JC, compared to the standard JC and participants perceived a number of the components of the TREAT format to be helpful in promoting skill development and engagement in the JC. Even so, quantitative data was unable to demonstrate that the format was more effective in improving individuals’ knowledge and skills in EBP compared with a standard JC. Rather, intermediate level EBP skills were maintained across both groups over time.

While previous studies have reported that JCs can increase clinicians’ knowledge and skills in EBP [3, 6–8], other studies which report some discrepancies in results [18, 21] may give insight into the lack of change to quantitative outcome measures in the present study. For example, an absence of quantitative change in EBP competency was reported in a previous study also using the ACE tool [21]. Consistent with our findings, Ilic et al., [21] found a disparity between positive qualitative data supporting an EBP intervention, and a lack of any significant quantitative difference. It was suggested that this lack of quantitative change may have been related to the fact that the majority of items in the ACE tool evaluate “cognitive knowledge rather than direct application in a clinical context” ([21] p 7). It is therefore possible that the ACE tool may not have been sensitive in detecting changes following JC.

Another possible explanation for the non-significant quantitative changes may be related to the length of the JC intervention. While previous JC research has used a similar 6 session format across 6 months [7], the present study’s participants had an average attendance of approximately 4 sessions. As such, it is possible that the intervention was not long enough to demonstrate a significant effect on knowledge and skills despite the qualitative reports of improved confidence. The finding that participants in the TREAT and standard JCs self-rated very positively on attitudes to EBP at pre-assessment may have also reduced the likelihood of significant changes post treatment from being identified on the EBPQ, as also reported by Lizarondo et al. [7].

The inclusion of nested focus groups and qualitative survey questions provided valuable evidence as to the feasibility of this evidence-based intervention in a clinical setting. Clinicians reported improved skills in appraisal and critical thinking as a result of JC participation, which is consistent with previous research [22]. Similarly to other studies, this study supported existing barriers to JC implementation such as heavy clinical workloads, staff changes and reduced skills [14, 22]. Due to many areas within allied health being research emergent, at times the only available evidence to answer a clinical question is of low quality. This lack of high quality evidence appraised within the JC was a novel barrier reported by allied health clinicians participating in journal clubs.

### Implications for practice

We identified evidence-based components of JCs that may enhance clinicians’ satisfaction. In particular, the use of an academic facilitator was seen by clinicians as both a source of expert knowledge and useful for keeping the flow of discussions in the JC. The collaborative group appraisal approach was perceived as valuable in promoting more active participation and reducing the time and responsibility of the presenting clinician. Seeking librarians’ help with searching, shared initial topic selection, and using structured critical appraisal tools were also seen as beneficial strategies. These may be important components for the practical implementation of JCs in other contexts. Qualitative findings support the feasibility of implementing JCs to enhance clinician engagement and maintain clinicians’ intermediate knowledge and skills in EBP, however, clinicians may benefit from additional training and strategies to enhance EBP skills and apply robust research findings to clinical practice.

### Limitations and implications for research

The current practice of rotating allied health staff throughout the hospital reduced the number of participants who were consistently able to attend JCs and complete both pre- and post-assessments. Even so, post hoc power calculations revealed a sample of 40 per group would still be able to detect a statistically significant difference of 6.1 points on the EBPQ which would be considered a minimum meaningful difference. It is therefore unlikely that the drop out led to insufficient statistical power. While the TREAT format and standard format were mostly different, some of the aspects of the TREAT format may have been used in the standard group (i.e., consistent time of meeting, discussion of application of the evidence). This overlap of components may have consequently made it more difficult to detect differences between groups.

Similar pragmatic trials, if conducted in other health settings, could help deepen our understanding of the effectiveness and feasibility of JCs and may contribute to a future meta-analysis. Further understanding is also required of the comparative contributions of each of these key components to JCs. It will also be important to ascertain whether current self-reported questionnaires and objective measures of EBP are sufficiently sensitive to detect change in the clinical application of research evidence. Future research may wish to explore the impact of the TREAT format compared to TREAT supplemented with further EBP education, or with other active EBP interventions potentially over a longer intervention period with longer follow up of the application of EBP skills over time. Reliable methods for measuring changes to clinical practice arising from journal club participation are also indicated.

## Conclusions

We demonstrated that it is feasible to implement an evidence-based structured JC for allied health clinicians that maintains their positive attitudes and intermediate EBP knowledge and skills. Participants in both groups reported a positive influence on their clinical practice. While EBP knowledge, skills, attitudes and practice did not improve in the structured TREAT journal club, compared to a standard journal club, participants were significantly more satisfied with the organisation of the TREAT JC. Qualitative findings supported the evidence base for effective JCs in allied health to promote skill development and engagement and it is suggested that JCs include the use of an academic facilitator, collaborative group based critical appraisal, and structured appraisal tools. While a structured JC may maintain EBP skills in allied health clinicians and facilitate engagement, additional training may be required to further enhance EBP skills and subsequent utilisation of evidence into clinical practice.

## Additional files

Additional file 1: This table outlines the session structure of the TREAT journal club format. (DOC 57 kb)

Additional file 2: This file shows a copy of the original questionnaire given to participants to measure their satisfaction with the journal club they participated it and its influence on their clinical practice. (DOC 44 kb)

Additional file 3: This file includes the interview guide used in the post treatment focus group for the TREAT participants. (DOC 24 kb)

Additional file 4: This table outlines each of the topics that were discussed in each of the journal club sessions. (DOC 40 kb)

Additional file 5: This table provides the frequency of individual item responses from the ACE tool both pre and post intervention. (DOC 60 kb)

Additional file 6: This table describes the reported frequency of changes to clinical practice following journal club reported by clinicians. (DOC 41 kb)

## Abbreviations

ACE tool: Assessing Competence in EBM tool
EBP: Evidence based practice
GCH: Gold Coast Health
JC: Journal club
TREAT: Tailoring Research Evidence and Theory
