# Supplementary material for: The effectiveness and feasibility of TREAT (Tailoring Research Evidence and Theory) journal clubs in allied health: a randomised controlled trial
Source: BMC Med Educ. 2018 May 9;18:104. doi: 10.1186/s12909-018-1198-y (PMC5944169; doi:10.1186/s12909-018-1198-y)
Supplement: Supplementary file 1 — This table outlines the session structure of the TREAT journal club format. (DOC 57 kb) [file 12909_2018_1198_MOESM1_ESM.doc]

# General session format: TREAT groups

**Resources needed for session**

- Critical appraisal sheets and minutes for scribe
- Journal article copies (these will be circulated by presenting clinician prior to session)
- Computer with internet access for power point presentation plus print outs
- Snacks (clinicians share bringing each month)

**Proposed frequency of sessions**

1 hour, once a month, 6 x sessions

**Facilitator: Academic mentor**

**General session format: WEEK 1:**

| **Activity** | **Detail:** | **Timeframe** |
| --- | --- | --- |
| **Welcome, overview of TREAT roles:** | Welcome everyone, introduce self again  *Academic Mentor & Facilitator:* Keeps group focussed and facilitates discussion as needed, able to provide expertise and support as required throughout the group, as well as identify whether any potential gaps could lead to potential research projects.  *Presenting clinician(s):* Rotating clinician(s) who present clinical question and accesses article with support from the Library. This can be done individually or in pairs. Nominated those for each six weeks.  *Scribe:* Documents clinical question, PICO, and decisions/actions made within group and saves electronically on an agreed location accessible to all members. Agree together where this will be? Allocation of scribe for today’s session? Provide template. | **5min** |
| **Initial didactic training** | Provided by academic mentor on prescribed topic  *Week 1:* forming a clinical PICO question  Powerpoint or handout given to members. | **10min** |
| **Clinical Question** | Clinical question (in line with original goals of group) introduced by presenting clinician(s) *(Clinician allocated to question accesses library for assistance with search prior to meeting to identify article).* | **5min** |
| **PICO & search strategy** | Process of forming PICO and search strategy for identifying article discussed (i.e., did the PICO need to be modified, what were search terms, what databases were searched) | **2-3min** |
| **Abstract reading & Appraisal** | Everyone reads the abstract independently  Article is appraised as a group using critical appraisal tool (CASP- Section A and B), everyone has copy of CASP, for larger groups or to increase involvement, break up into groups of 2-3 and look at different questions of the tool (e.g., pairs do 1 questions each and then come back to discuss after a few minutes).  Note: Section B interpretation of results is often more challenging for clinicians and may require more guidance from mentor initially. | **20min** |
| **Application to clinical context** | Review evidence in terms of application to clinical context, patient values and experience (as per CASP- Section C). | **10min** |
| **Follow up** | Discuss any follow up required regarding implementation of evidence and who is accountable (to be followed up at subsequent session) which is documented by the scribe | **5min** |

**General session format: WEEKS 2-5**

| **Activity** | **Detail:** | **Timeframe** |
| --- | --- | --- |
| **Actions from last meeting** | Nominate scribe for the session.  Follow up previous actions from last meeting and troubleshoot any barriers. Modify new actions if needed (documented by scribe). | **5min** |
| **Sharing of resources** | (THIS IS NOW GIVEN AS HANDOUT) Provided by academic mentor on prescribed topic  Week 2- basics of appraisal/ types of bias.  Week 3- searching the literature  Week 4- statistics (data types and comparing numbers)  Week 5- basic statistics (clinical vs statistical significance)  Powerpoint or handout given to members. | **10min** |
| **Clinical Question** | Clinical question (in line with original goals of group) introduced by presenting clinician(s)  *(Clinician allocated to question accesses library for assistance with search prior to meeting to identify article).* | **5min** |
| **PICO & search strategy** | Process of forming PICO and search strategy for identifying article discussed (i.e., did the PICO need to be modified, what were search terms, what databases were searched) | **2-3min** |
| **Abstract reading & Appraisal** | Everyone reads the abstract independently  Article is appraised as a group using critical appraisal tool (CASP- Section A and B), everyone has copy of CASP, for larger groups or to increase involvement, break up into groups of 2-3 and look at different questions of the tool (e.g., pairs do 1 questions each and then come back to discuss after a few minutes).  Note: Section B interpretation of results is often more challenging for clinicians and may require more guidance from mentor initially. | **20min** |
| **Application to clinical context** | Review evidence in terms of application to clinical context, patient values and experience (as per CASP- Section C). | **10min** |
| **Follow up** | Discuss any follow up required regarding implementation of evidence and who is accountable (to be followed up at subsequent session) which is documented by the scribe | **5min** |

**General session format:** WEEK 6

| **Activity** | **Detail:** | **Timeframe** |
| --- | --- | --- |
| **Actions from last meeting** | Nominate scribe for the session.  Follow up previous actions from last meeting and troubleshoot any barriers. Modify new actions if needed (documented by scribe). | **5min** |
| **Initial didactic training** | Provided by academic mentor on prescribed topic  Week 6- basic statistics (diagnostic studies)  Powerpoint or handout given to members. | **8-10min** |
| **Clinical Question** | Clinical question (in line with original goals of group) introduced by presenting clinician(s)  *(Clinician allocated to question accesses library for assistance with search prior to meeting to identify article).* | **5min** |
| **PICO & search strategy** | Process of forming PICO and search strategy for identifying article discussed (i.e., did the PICO need to be modified, what were search terms, what databases were searched) | **2min** |
| **Abstract reading & Appraisal** | Everyone reads the abstract independently  Article is appraised as a group using critical appraisal tool (CASP- Section A and B), everyone has copy of CASP, for larger groups or to increase involvement, break up into groups of 2-3 and look at different questions of the tool (e.g., pairs do 1 questions each and then come back to discuss after a few minutes).  Note: Section B interpretation of results is often more challenging for clinicians and may require more guidance from mentor initially. | **20min** |
| **Application to clinical context** | Review evidence in terms of application to clinical context, patient values and experience (as per CASP- Section C). | **10min** |
| **Follow up** | Discuss any follow up required regarding implementation of evidence and who is accountable (to be followed up at subsequent session) which is documented by the scribe. | **5min** |
| **Wrap up** | Describe this is final formal session of trial where you will be facilitated by an academic mentor and next 6 months you are welcome to continue this format or adapt it to however you see fit. Its up to you as a group.  In 6 months time you will have another assessment to see how the impact of the journal club on your EBP skills and also whether the format was sustainable or not. | **5min** |
