# Supplementary material for: The effectiveness and feasibility of TREAT (Tailoring Research Evidence and Theory) journal clubs in allied health: a randomised controlled trial
Source: BMC Med Educ. 2018 May 9;18:104. doi: 10.1186/s12909-018-1198-y (PMC5944169; doi:10.1186/s12909-018-1198-y)
Supplement: Supplementary file 2 — This file shows a copy of the original questionnaire given to participants to measure their satisfaction with the journal club they participated it and its influence on their clinical practice. (DOC 44 kb) [file 12909_2018_1198_MOESM2_ESM.doc]

**Satisfaction Questionnaire:**

Have you taken part in any formal EBP training in the last 6 months?

YES NO

If yes, please indicate the duration of this training (in hours)

___________________________________________________

Please rate your level of agreement with the following statements in regards to the journal club you have participated in over the last 6 months:

*I found the topics discussed in the journal club were useful to my clinical practice*

1______________2______________3________________4________________5

Strongly Disagree Neutral Agree Strongly

Disagree Agree

*I think attending this journal club is a valuable use of my time*

1______________2______________3________________4________________5

Strongly Disagree Neutral Agree Strongly

Disagree Agree

The journal club format was overall well organised

1______________2______________3________________4________________5

Strongly Disagree Neutral Agree Strongly

Disagree Agree

The journal club format I participated in should continue

1______________2______________3________________4________________5

Strongly Disagree Neutral Agree Strongly

Disagree Agree

*Overall, I would recommend participation in the journal club to other clinicians*

1______________2______________3________________4________________5

Strongly Disagree Neutral Agree Strongly

Disagree Agree

What did you find most useful about the Journal Club you participated in over the last 6 months?

____________________________________________________________________________________________________________________________________________________________________________________________________________________________________

What factors (if any) do you believe helped to facilitate the effectiveness of the journal club?

____________________________________________________________________________________________________________________________________________________________________________________________________________________________________

What (if any) barriers did you encounter which you believe hindered the effectiveness of the journal club?

____________________________________________________________________________________________________________________________________________________________________________________________________________________________________

________________________________________________________

How do you think the Journal club format that you participated over the last 6 months could be improved in the future?

____________________________________________________________________________________________________________________________________________________________________________________________________________________________________

**Influence of Journal club on clinical practice**

The following questions are based on topics that you reviewed as part of your journal club over the last six months.

**Topic 1:** (*Journal club topic inserted here)*

*Please tick one:*

- I did not attend this journal club (go to Topic 2)
- I attended this journal club but the topic was not relevant to me (go to Topic 2)
- I attended this journal club and the topic was relevant to me (please continue)

**Please rate ONE of the following statements:**

1. *The article discussed suggested I do not need to change my practice as what I currently do was supported by the evidence*

1______________2______________3________________4________________5

Strongly Disagree Neutral Agree Strongly

Disagree Agree

**OR**

1. *The article discussed provided new evidence that changed how I will/do provide clinical services to patients.*

1______________2______________3________________4________________5

Strongly Disagree Neutral Agree Strongly

Disagree Agree

**Did the discussion at the journal club lead to any of the following changes for your service?**

- Updated guideline, process or pathway
- Adopted new treatment strategy or resource
- Stopped current practice due to lack of evidence
- Commenced new research project
- Commenced new quality project

Other (please indicated)

_________________________________________________________________________________________________________________________________________________________________________________________________________

**Topic 2:** (*Journal club topic inserted here)*

*Please tick one:*

- I did not attend this journal club (go to Topic 3)
- I attended this journal club but the topic was not relevant to me (go to Topic 3)
- I attended this journal club and the topic was relevant to me (please continue)

**Please rate ONE of the following statements:**

1. *The article discussed suggested I do not need to change my practice as what I currently do was supported by the evidence*

1______________2______________3________________4________________5

Strongly Disagree Neutral Agree Strongly

Disagree Agree

**OR**

1. *The article discussed provided new evidence that changed how I will/do provide clinical services to patients.*

1______________2______________3________________4________________5

Strongly Disagree Neutral Agree Strongly

Disagree Agree

**Did the discussion at the journal club lead to any of the following changes for your service?**

- Updated guideline, process or pathway
- Adopted new treatment strategy or resource
- Stopped current practice due to lack of evidence
- Commenced new research project
- Commenced new quality project

Other (please indicated)

_________________________________________________________________________________________________________________________________________________________________________________________________________

**Topic 3:** (*Journal club topic inserted here)*

*Please tick one:*

- I did not attend this journal club (go to Topic 4)
- I attended this journal club but the topic was not relevant to me (go to Topic 4)
- I attended this journal club and the topic was relevant to me (please continue)

**Please rate ONE of the following statements:**

1. *The article discussed suggested I do not need to change my practice as what I currently do was supported by the evidence*

1______________2______________3________________4________________5

Strongly Disagree Neutral Agree Strongly

Disagree Agree

**OR**

1. *The article discussed provided new evidence that changed how I will/do provide clinical services to patients.*

1______________2______________3________________4________________5

Strongly Disagree Neutral Agree Strongly

Disagree Agree

**Did the discussion at the journal club lead to any of the following changes for your service?**

- Updated guideline, process or pathway
- Adopted new treatment strategy or resource
- Stopped current practice due to lack of evidence
- Commenced new research project
- Commenced new quality project

Other (please indicated)

_________________________________________________________________________________________________________________________________________________________________________________________________________

**Topic 4:** (*Journal club topic inserted here)*

*Please tick one:*

- I did not attend this journal club (go to Topic 5)
- I attended this journal club but the topic was not relevant to me (go to Topic 5)
- I attended this journal club and the topic was relevant to me (please continue)

**Please rate ONE of the following statements:**

1. *The article discussed suggested I do not need to change my practice as what I currently do was supported by the evidence*

1______________2______________3________________4________________5

Strongly Disagree Neutral Agree Strongly

Disagree Agree

**OR**

1. *The article discussed provided new evidence that changed how I will/do provide clinical services to patients.*

1______________2______________3________________4________________5

Strongly Disagree Neutral Agree Strongly

Disagree Agree

**Did the discussion at the journal club lead to any of the following changes for your service?**

- Updated guideline, process or pathway
- Adopted new treatment strategy or resource
- Stopped current practice due to lack of evidence
- Commenced new research project
- Commenced new quality project

Other (please indicated)

_________________________________________________________________________________________________________________________________________________________________________________________________________

**Topic 5:** (*Journal club topic inserted here)*

*Please tick one:*

- I did not attend this journal club (go to Topic 6)
- I attended this journal club but the topic was not relevant to me (go to Topic 6)
- I attended this journal club and the topic was relevant to me (please continue)

**Please rate ONE of the following statements:**

1. *The article discussed suggested I do not need to change my practice as what I currently do was supported by the evidence*

1______________2______________3________________4________________5

Strongly Disagree Neutral Agree Strongly

Disagree Agree

**OR**

1. *The article discussed provided new evidence that changed how I will/do provide clinical services to patients.*

1______________2______________3________________4________________5

Strongly Disagree Neutral Agree Strongly

Disagree Agree

**Did the discussion at the journal club lead to any of the following changes for your service?**

- Updated guideline, process or pathway
- Adopted new treatment strategy or resource
- Stopped current practice due to lack of evidence
- Commenced new research project
- Commenced new quality project

Other (please indicated)

_________________________________________________________________________________________________________________________________________________________________________________________________________

**Topic 6:** (*Journal club topic inserted here)*

*Please tick one:*

- I did not attend this journal club (go to next section)
- I attended this journal club but the topic was not relevant to me (go to next section)
- I attended this journal club and the topic was relevant to me (please continue)

**Please rate ONE of the following statements:**

1. *The article discussed suggested I do not need to change my practice as what I currently do was supported by the evidence*

1______________2______________3________________4________________5

Strongly Disagree Neutral Agree Strongly

Disagree Agree

**OR**

1. *The article discussed provided new evidence that changed how I will/do provide clinical services to patients.*

1______________2______________3________________4________________5

Strongly Disagree Neutral Agree Strongly

Disagree Agree

**Did the discussion at the journal club lead to any of the following changes for your service?**

- Updated guideline, process or pathway
- Adopted new treatment strategy or resource
- Stopped current practice due to lack of evidence
- Commenced new research project
- Commenced new quality project

Other (please indicated)

_________________________________________________________________________________________________________________________________________________________________________________________________________

**TOTAL SCORE (Researcher use only) = ___/30**
