# Supplementary material for: The effectiveness and feasibility of TREAT (Tailoring Research Evidence and Theory) journal clubs in allied health: a randomised controlled trial
Source: BMC Med Educ. 2018 May 9;18:104. doi: 10.1186/s12909-018-1198-y (PMC5944169; doi:10.1186/s12909-018-1198-y)
Supplement: Supplementary file 3 — This file includes the interview guide used in the post treatment focus group for the TREAT participants. (DOC 24 kb) [file 12909_2018_1198_MOESM3_ESM.doc]

**Interview guide FOCUS group:**

- How would you describe your experience with participating in the TREAT journal club format?
- How would you compare the TREAT journal club with other more traditional journal club formats you have participated in previously?
- *{Facilitator to show list of core components of TREAT club to members} to discuss*. In your experience, what components of the TREAT Journal club do you think were most useful? (and why)
- What components of the TREAT Journal club do you think were least useful? (and why)
- What barriers (if any) did you encounter which may have hindered the effectiveness of the journal club?
- What factors (if any) do you believe may have helped the effectiveness of the journal club?
- Are there any examples of how participation in the TREAT journal club influenced your clinical practice?
- Is there anything you would do differently to the TREAT Journal club format?
- How sustainable do you think this journal club format will be in your current setting?
