# Supplementary material for: The effectiveness and feasibility of TREAT (Tailoring Research Evidence and Theory) journal clubs in allied health: a randomised controlled trial
Source: BMC Med Educ. 2018 May 9;18:104. doi: 10.1186/s12909-018-1198-y (PMC5944169; doi:10.1186/s12909-018-1198-y)
Supplement: Supplementary file 4 — This table outlines each of the topics that were discussed in each of the journal club sessions. (DOC 40 kb) [file 12909_2018_1198_MOESM4_ESM.doc]

Additional File 4: Journal club topics each session

| **Journal club/**  **Group** | **Session topic each week** | | | | | |
| --- | --- | --- | --- | --- | --- | --- |
|  | **Session 1** | **Session 2** | **Session 3** | **Session 4** | **Session 5** | **Session 6** |
| **TREAT**  **JC 1** | Cognitive sensory motor  training therapy for arm function in acute stroke patients | OT treatment with right half-field eye-patching for subacute stroke and unilateral neglect | Training of reaching in stroke survivors with severe and chronic upper limb paresis using a novel non robotic device | Shared decision making within goal setting in rehabilitation settings | An enriched environment increases activity in stroke patients undergoing rehab in a mixed rehabilitation unit | The effect of occupation-based cognitive rehabilitation  for traumatic brain injury |
| **TREAT**  **JC 2** | Recommendations for optimal dietary protein intake in older people | Prophylactic phosphate supplementation for the inpatient treatment of restrictive eating disorders | Refeeding enteroclysis as an alternative to parenteral nutrition for enteric fistula | The effectiveness of a specialised oral nutrition supplement in patients with chronic wounds | The prevalence of weight loss during (chemo) radiotherapy treatment for lung cancer and associated patient- and treatment-related factors | Does adding a dietitian to the liaison team after discharge of geriatric patients improve nutritional outcome |
| **TREAT JC 3** | Improved self-management skills predict improvements in QoL and depression in patients with chronic disorders | Empagliflozin, Cardiovascular Outcomes and Mortality in Type 2 Diabetes | MRC/BHF Heart Protection Study of Cholesterol-lowing with simvastin in diabetes | Adherence to exercise programs for older people is influenced by program characteristics and personal factors | What is the effect of peer support on diabetes outcomes in adults | Spironolactone for Heart Failure with Preserved Ejection Fraction |
| **TREAT JC 4** | Early mobilisation after stroke | Requirement for 100% oxygen before and after closed suction | Instruments for assessing the risk of falls in acute hospitalised patients | Effects of an acute care for elders unit on costs and 30 day readmissions | Stretch for the treatment and prevention of contractures | Effect of primary care based education on reassurance in patients with lower back pain |
| **TREAT JC 5** | Promoting independence in frail older people | Patients undergoing subacute rehabilitation and expectations of their health related QOL at discharge | Effectiveness of motivational interviewing after acute stroke | Integrated multidisciplinary osteoarthritis outpatient clinic versus outpatient clinic as usual | Increasing patient insight after stroke | Measuring relational aspects of hospital care |
| **Standard JC 1** | Dynamic hyperinflation during activities of daily living in COPD | Balance and Eye Movement Training to Improve Gait in Progressive Supranuclear Palsy | Swallow related QOL in ALS | Discontinuation of Inhaled Corticosteroids in COPD and the Risk Reduction of Pneumonia | Direct inpatient burden caused by foot-related conditions: a multisite point-prevalece study | Becoming a Clinician Researcher in Allied Health |
| **Standard JC 2** | Task-specific training | Individuals with stroke reporting unmet need for OT following discharge | OT rehab interventions with deconditioned older adults | No topic | The use of MoCA within an in-patient rehab setting | No topic |
| **Standard JC 3** | A predictive model for diagnosing stroke-related apraxia of speech.  Oropharyngeal dysphagia: the experience of patients with non-head and neck cancers receiving specialist palliative care. | Speech-language pathologists’ contribution to the assessment of decision-making capacity in aphasia  AND  New Intensive Speech Treatment for Adults with Non progressive Dysarthria | A Retrospective Review of Swallow Dysfunction in Patients with Severe Traumatic Brain Injury  AND  The Comprehensive Aphasia Test | Differential effects of premorbid physical and emotional health on recovery from acute events.  AND  Older people with dysphagia: transitioning to texture-modified food | Preliminary data on two voice therapy interventions in the treatment of presbyphonia  AND  Insight and awareness in a rehabilitation patient | Effect of Verb Network Strengthening Treatment in Persons With  Aphasia: Extension and Replication of Previous Findings |
| Standard JC 4 | Flight 1 and Flight 2 – Indacaterol/Glycopyrronium for COPD treatment | HALLMARK-DUAL trial – Hepatitis C novel direct acting oral regimen | Association of PPIs with Risk of Dementia | Pharmavcovigilance in hospice/palliative care: the net immediate and short-term effects of dexamethasone for anorexia | Empagliflozin, Cardiovascular Outcomes, and Mortality in Type 2 Diabetes | Olanzapine for the Prevention of  Chemotherapy-Induced Nausea and Vomiting |

N.B= COPD= chronic obstructive pulmonary disease, QOL= quality of life, ALS= amyolateral sclerosis, OT= occupational therapy
