# Supplementary material for: The effectiveness and feasibility of TREAT (Tailoring Research Evidence and Theory) journal clubs in allied health: a randomised controlled trial
Source: BMC Med Educ. 2018 May 9;18:104. doi: 10.1186/s12909-018-1198-y (PMC5944169; doi:10.1186/s12909-018-1198-y)
Supplement: Supplementary file 5 — This table provides the frequency of individual item responses from the ACE tool both pre and post intervention. (DOC 60 kb) [file 12909_2018_1198_MOESM5_ESM.doc]

| Individual item responses from ACE tool pre- and post-intervention | | | | | | | | |  |  |  |  |  |  |  |  |
| --- | --- | --- | --- | --- | --- | --- | --- | --- | --- | --- | --- | --- | --- | --- | --- | --- |
|  | Answers provided pre- and post-intervention (*N* TREAT = 41, Standard = 39) | | | | | | | | | | | | | | | |
| ACE Items | **Right-Right** | | | | **Right-Wrong** | | | | **Wrong-Right** | | | | **Wrong-Wrong** | | | |
|  | TREAT | | Standard | | TREAT | | Standard | | TREAT | | Standard | | TREAT | | Standard | |
|  | Freq | % | Freq | % | Freq | % | Freq | % | Freq | % | Freq | % | Freq | % | Freq | % |
| **Answerable Question** |  |  |  |  |  |  |  |  |  |  |  |  |  |  |  |  |
| PICO elements described | 18 | 43.6 | 18 | 46.2 | 6 | 14.6 | 6 | 15.4 | 10 | 24.4 | 10 | 25.6 | 7 | 17.1 | 5 | 12.8 |
| Focused question | 17 | 41.5 | 10 | 25.6 | 8 | 19.5 | 12 | 30.8 | 3 | 7.3 | 8 | 20.5 | 13 | 31.7 | 9 | 23.1 |
| **Searching the Literature** |  |  |  |  |  |  |  |  |  |  |  |  |  |  |  |  |
| Relevant search strategy | 26 | 63.4 | 24 | 37.5 | 2 | 4.9 | 6 | 15.4 | 8 | 19.5 | 5 | 12.8 | 5 | 12.2 | 4 | 10.3 |
| Boolean operators | 19 | 46.3 | 10 | 25.6 | 4 | 9.8 | 8 | 20.5 | 12 | 29.3 | 10 | 25.6 | 6 | 14.6 | 11 | 28.2 |
| **Appraising the Evidence** |  |  |  |  |  |  |  |  |  |  |  |  |  |  |  |  |
| Representativeness | 22 | 53.7 | 26 | 66.7 | 7 | 17.1 | 5 | 12.8 | 8 | 19.5 | 7 | 17.9 | 4 | 9.8 | 1 | 2.6 |
| Allocation method | 6 | 14.6 | 0 | 0 | 0 | 0 | 3 | 7.7 | 3 | 7.3 | 5 | 12.8 | 32 | 78 | 31 | 79.5 |
| Adjustment required | 11 | 26.8 | 14 | 35.9 | 7 | 17.1 | 7 | 17.9 | 12 | 29.3 | 8 | 20.5 | 11 | 26.8 | 10 | 25.6 |
| Participants blinded | 39 | 98.1 | 34 | 53.1 | 0 | 0 | 1 | 2.6 | 2 | 4.9 | 4 | 10.3 | 0 | 0 | 0 | 0 |
| Investigators blinded | 30 | 73.2 | 28 | 71.8 | 4 | 9.8 | 3 | 7.7 | 5 | 12.2 | 7 | 17.9 | 2 | 4.9 | 1 | 2.6 |
| Assessors blinded | 31 | 75.6 | 26 | 66.7 | 3 | 7.3 | 4 | 10.3 | 7 | 17.1 | 7 | 17.9 | 0 | 0 | 2 | 5.1 |
| Intention to treat | 3 | 7.3 | 4 | 10.3 | 5 | 12.2 | 9 | 23.1 | 5 | 12.2 | 2 | 5.1 | 28 | 68.5 | 24 | 61.5 |
| **Applying the Evidence** |  |  |  |  |  |  |  |  |  |  |  |  |  |  |  |  |
| Scenario similar to study | 29 | 70.7 | 29 | 74.4 | 2 | 4.9 | 2 | 5.1 | 6 | 14.6 | 6 | 15.4 | 4 | 9.8 | 2 | 5.1 |
| Feasible treatment | 32 | 78 | 29 | 74.4 | 2 | 4.9 | 3 | 7.7 | 7 | 17.1 | 4 | 10.3 | 0 | 0 | 3 | 7.7 |
| Clinically important outcomes | 7 | 17.1 | 12 | 30.8 | 19 | 46.3 | 8 | 20.5 | 3 | 7.3 | 3 | 7.7 | 12 | 29.3 | 16 | 41 |
| Benefits/harms | 4 | 9.8 | 4 | 10.3 | 10 | 24.4 | 6 | 15.4 | 1 | 2.4 | 2 | 5.1 | 26 | 63.4 | 27 | 69.2 |
| Note. TREAT = TREAT Journal Club; Freq = frequency of responses | | | | | | | | | | | | | | | | |
