# Supplementary material for: The effectiveness and feasibility of TREAT (Tailoring Research Evidence and Theory) journal clubs in allied health: a randomised controlled trial
Source: BMC Med Educ. 2018 May 9;18:104. doi: 10.1186/s12909-018-1198-y (PMC5944169; doi:10.1186/s12909-018-1198-y)
Supplement: Supplementary file 6 — This table describes the reported frequency of changes to clinical practice following journal club reported by clinicians. (DOC 41 kb) [file 12909_2018_1198_MOESM6_ESM.doc]

**Reported frequency of changes to clinical practice following journal club reported by clinicians**

| **Week** | **Group** | **Updated guideline** | **Adopted New therapy** | **Stopped existing therapy** | **Led to Research Activity** | **Led to QA Activity** |
| --- | --- | --- | --- | --- | --- | --- |
| **1** | TREAT  (n=32) | 3 | 4 | 1 | 0 | 0 |
|  | STANDARD  (n=31) | 2 | 9 | 1 | 0 | 0 |
| **2** | TREAT  (n=27) | 3 | 4 | 0 | 0 | 0 |
|  | STANDARD  (n=24) | 2 | 6 | 0 | 0 | 2 |
| **3** | TREAT  (n=23) | 2 | 4 | 1 | 1 | 0 |
|  | STANDARD  (n=29) | 3 | 7 | 1 | 0 | 0 |
| **4** | TREAT  (n=23) | 0 | 5 | 1 | 0 | 1 |
|  | STANDARD  (n=24) | 2 | 5 | 0 | 0 | 0 |
| **5** | TREAT  (n=20) | 5 | 5 | 2 | 0 | 2 |
|  | STANDARD  (n=28) | 2 | 7 | 1 | 0 | 1 |
| **6** | TREAT (n=29) | 2 | 1 | 0 | 1 | 1 |
|  | STANDARD  (n=24) | 2 | 7* | 0 | 0 | 0 |
| **TOTAL** | **TREAT** | **15** | **23** | **5** | **2** | **4** |
|  | **STANDARD** | **13** | **36** | **3** | **0** | **3** |

*= significant difference between groups
